# *De novo* sequencing and comparative analysis of testicular transcriptome from different reproductive phases in freshwater spotted snakehead *Channa punctatus*

**DOI:** 10.1371/journal.pone.0173178

**Published:** 2017-03-02

**Authors:** Alivia Roy, Reetuparna Basak, Umesh Rai

**Affiliations:** Department of Zoology, University of Delhi, Delhi, India; University of Hyderabad, INDIA

## Abstract

The spotted snakehead *Channa punctatus* is a seasonally breeding teleost widely distributed in the Indian subcontinent and economically important due to high nutritional value. The declining population of *C*. *punctatus* prompted us to focus on genetic regulation of its reproduction. The present study carried out *de novo* testicular transcriptome sequencing during the four reproductive phases and correlated differential expression of transcripts with various testicular events in *C*. *punctatus*. The Illumina paired-end sequencing of testicular transcriptome from resting, preparatory, spawning and postspawning phases generated 41.94, 47.51, 61.81 and 44.45 million reads, and 105526, 105169, 122964 and 106544 transcripts, respectively. Transcripts annotated using *Rattus norvegicus* reference protein sequences and classified under various subcategories of biological process, molecular function and cellular component showed that the majority of the subcategories had highest number of transcripts during spawning phase. In addition, analysis of transcripts exhibiting differential expression during the four phases revealed an appreciable increase in upregulated transcripts of biological processes such as cell proliferation and differentiation, cytoskeleton organization, response to vitamin A, transcription and translation, regulation of angiogenesis and response to hypoxia during spermatogenically active phases. The study also identified significant differential expression of transcripts relevant to spermatogenesis (*mgat3*, *nqo1*, *hes2*, *rgs4*, *cxcl2*, *alcam*, *agmat*), steroidogenesis (*star*, *tkt*, *gipc3*), cell proliferation (*eef1a2*, *btg3*, *pif1*, *myo16*, *grik3*, *trim39*, *plbd1*), cytoskeletal organization (*espn*, *wipf3*, *cd276*), sperm development (*klhl10*, *mast1*, *hspa1a*, *slc6a1*, *ros1*, *foxj1*, *hipk1*), and sperm transport and motility (*hint1*, *muc13*). Analysis of functional annotation and differential expression of testicular transcripts depending on reproductive phases of *C*. *punctatus* helped in developing a comprehensive understanding on genetic regulation of spermatogenic and steroidogenic events in seasonally breeding teleosts. Our findings provide the basis for future investigation on the precise role of testicular genes in regulation of seasonal reproduction in male teleosts.

## Introduction

Spermatogenesis is an exquisitely orchestrated developmental process during which temporal expression of various testicular genes regulate proliferation and differentiation of diploid spermatogonia to give rise to haploid spermatozoa [1–4]. Nonetheless, reports on molecular control of testicular functions are largely confined to continuous breeders and little attention has been paid to discontinuous breeders in which testis undergoes cyclical changes from inactive to active state depending on season. Among seasonally breeding vertebrates, fishes comprise the largest and most economically important group contributing fifteen percent of average animal protein intake per person for more than 4.3 billion people in the world [5]. In spite of that most of the studies in fishes to comprehend genetic basis of spermatogenesis are restricted to identifying testicular genes [6–18] and only a few reports are focused on differential expression of genes along the testicular cycle [19,20]. The various techniques adopted in these studies were cDNA microarray, EST sequencing, subtractive and suppressive hybridization (SSH), and RNA sequencing (RNA-Seq). Among these, RNA-Seq is the most efficient and cost-effective technique enabling high-throughput sequencing of the entire transcriptome at single-base resolution and accurate quantification of gene expression [21].

In the present study, RNA-Seq using Illumina platform was employed to obtain testicular transcriptome of different reproductive phases from a seasonally breeding freshwater teleost spotted snakehead *Channa punctatus* belonging to family Channidae and order Perciformes. Fishes of this family constitute one of the major component of pond fishery in the Indian subcontinent and are economically important due to their high nutritional and medicinal value [22]. This species of *Channa* has been enlisted under the Lower Risk near threatened category due to its declining population [23] and hence, it is important to gain an insight on the genetic regulation of reproduction in this fish. Efforts have been made in the current study to obtain *de novo* testicular transcriptome from different reproductive phases and develop a comprehensive understanding of temporal expression of genes implicated in regulation of spermatogenesis in *C*. *punctatus*.

## Methods

### Ethics statement

As per guidelines of the Committee for the Purpose of Control and Supervision of Experiments on Animals (CPCSEA), Government of India, the detailed protocol of this study was approved by the Institutional Animal Ethics Committee, Department of Zoology (DUZOOL/IAEC-R/2012/20), University of Delhi, India. To euthanize fishes, 2-phenoxyethanol was added in the water (5 ml per litre).

### Animals and tissue collection

The testicular cycle of *C*. *punctatus* obtained from Delhi and its vicinity has been delineated into four phases: resting (December to March), preparatory (April to June), spawning (July and August) and postspawning (September to November) [24]. During resting phase, fishes are spermatogenically inactive and their seminiferous lobules consist largely of spermatogonia and a few spermatogonial stem cells. Spermatogenesis commences during preparatory phase which is characterized by the presence of different stages of germ cells from spermatogonia to spermatozoa. Thereafter, during spawning phase, lumen of seminiferous lobules is packed with spermatozoa. Due to release of spermatozoa into the external environment, a few lobules with empty lumen are also seen in spawning phase. Subsequently, remnant germ cells undergo cell death and proliferation of spermatogonia is resumed in order to repopulate the seminiferous lobules during postspawning phase. In the present study, adult male spotted snakehead (100–120 g) captured from freshwater bodies of Delhi (latitude 28.38’N, longitude 77.20’E) and its vicinity were supplied during the mid of January (resting phase), May (preparatory phase), August (spawning phase) and October (postspawning phase) by a local vendor (Kalyanpuri, Delhi, India). Fishes were euthanized by overexposure to 2-phenoxyethanol (Loba Chemie, Mumbai, India) added in water (5 ml per litre). Testes dissected out from ten fishes were pooled to make one sample of 100 mg tissue weight and thus, two such samples were prepared for each reproductive phase. The samples from resting (sample R), preparatory (sample P), spawning (sample S) and postspawning (sample Ps) phases were frozen in liquid nitrogen and stored at -80°C prior to transcriptome sequencing carried out by Genotypic Technology Pvt. Ltd., Bengaluru, India.

### RNA extraction, cDNA library preparation and sequencing

One sample from each phase was processed for total RNA extraction with Trizol reagent (Thermo Fisher Scientific, Waltham, Massachusetts, USA) and RNeasy Mini Kit (Qiagen, Valencia, California, USA). RNA concentration and integrity were examined with Bioanalyzer. Samples having A_260_/A_280_ absorption ratios within range of 1.8–2.1 and RNA integrity number (RIN) 7 or above were selected for cDNA library preparation. The duplicate tissue sample was processed for RNA extraction only when its corresponding replicate failed to qualify the quality criteria. The protocol outlined in TruSeq RNA sample preparation guide (Illumina, Inc., San Diego, California, USA) was followed for constructing the library. Briefly, mRNA purified from 1 μg of total RNA using oligodT beads (TruSeq RNA Sample Preparation Kit, Illumina) was fragmented at 94°C for 4 min in the presence of divalent cations. Subsequently, mRNA was primed with random hexamers and reverse transcribed using Superscript II reverse transcriptase (Invitrogen, Waltham, Massachusetts, USA). The second cDNA strand was synthesized with DNA Polymerase I and RnaseH and the double-stranded cDNA was purified using solid phase reverse immobilization (SPRI) beads (AgencourtAMPure XP kit, Beckman Coulter, Brea, California, USA). After end repair and addition of base A, Illumina adapters were ligated to the cDNA followed by SPRI cleanup. Further, the adapter ligated fragments were amplified by 11 PCR cycles. The prepared library was quantified using Nanodrop and validated for quality using High Sensitivity Bioanalyzer Kit (Agilent technologies, Santa Clara, California, USA). Finally, Illumina paired-end transcriptome sequencing of the cDNA library was performed on HiSeq 2000 platform to obtain reads of 100 bp length followed by generation of FASTQ files using Illumina pipeline software.

### Data filtering and *de novo* assembly

Obtained FASTQ reads for each sample were subjected to quality check using Genotypic proprietary tool SeqQC- V2.1. Low quality bases (quality score < 20) were trimmed and adapter sequences were removed using custom perl codes. Thereafter, reads ranging between 50–100 bp were selected for *de novo* assembly into contigs (minimum length 100 bp) using Velvet (version 1.2.07) followed by generation of transcripts (minimum length 200 bp) using Oases (version 0.2.08) assembler. Also, transcripts generated from testicular samples of four reproductive phases (R, P, S and Ps) were clustered using CD-Hit tool [25] to obtain the total testicular transcriptome.

### Functional annotation of transcripts

Transcripts obtained from testicular sample of each reproductive phase were annotated based on the best hit of BLASTX results against reference protein sequences of *Rattus norvegicus*, *Oreochromis niloticus* and *Takifugu rubripes* available at Uniprot. Maximum number of transcripts in each reproductive phase was annotated against Uniprot *R*. *norvegicus* reference protein sequences, and hence, the same protein database was used for Gene Ontology (GO) annotation of total testicular transcriptome. Using NCBI-BLAST 2.2.28, the identified transcripts were assigned GO subcategories under biological process (BP), molecular function (MF) and cellular component (CC). Further, variation in transcript numbers of different subcategories depending on reproductive phases was analyzed.

### Differential gene expression analysis

For comparative analysis of genes expressed along the testicular cycle, four sets of clustered transcripts were generated from samples of different reproductive phases using CD-Hit at 95% identity/coverage (set 1: samples R and P; set 2: samples P and S; set 3: samples S and Ps; set 4: samples Ps and R). Further, differential gene expression (DGE) data was obtained for each set of clustered transcripts using DESeq software [26] and expression fold change was calculated considering the underlined sample in each set as reference. Transcripts having log2 (fold change) value ≥ 1 and ≤ -1 were considered to be upregulated and downregulated, respectively. Thereafter, up- and down-regulated transcripts along the different reproductive phases were grouped as per their GO subcategories. In addition, transcripts showing significant variation in expression fold change (corrected *P* value ≤ 0.05) in these sets were identified. Also, the expression level of Sertoli cell (SC), Leydig cell (LC) and peritubular myoid cell (PMC) specific genes during different phases of the testicular cycle were analyzed.

### Validation of differential gene expression

To validate the RNA-Seq data of differentially expressed testicular genes, expression of some of these genes enlisted in Table 1 was estimated during different reproductive phases by quantitative polymerase chain reaction (qPCR).

**Table 1:** Enlisting the selected genes and their primers for quantitative PCR.

Gene name	Gene accession number	Primer sequences
transketolase (***tkt***)	GEMA01054854.1 under TSA accession GEMA00000000	FP: 5’-GACCACTACCACGAAGG-3’
		RP: 5’-AGGAACGTGGGACACAG-3’
Mannosyl (beta-1,4-)-glycoprotein beta-1,4-N-acetylglucosaminyltransferase (***mgat3***)	GEMA01063365.1 under TSA accession GEMA00000000	FP: 5’-CTGGTAAAGTGTGTGTGCCG-3’
		RP: 5’-TTAGTGGGCAGGTTGGAGTGG-3’
Activated leukocyte cell adhesion molecule (***alcam***)	GEKZ01011742.1 under TSA accession GEKZ00000000	FP: 5’-CATGAAGAAGTCCAAACAAGG-3’
		RP: 5’-TTTTTGACTGTTCTCCTCCAC-3’
GIPC PDZ domain containing family member 3 (***gipc3***)	GEKY01111158.1 under TSA accession GEKY00000000	FP: 5’-TGACCAGAGCATTGTAGG-3’
		RP: 5’-CTAGGCGAAGAGTGAAG-3’
syntaxin 1B (***stx1b***)	GEKY01043631.1 under TSA accession GEKY00000000	FP: 5’-AATCGAACAGCGGCACAAGG-3’
		RP: 5’-CTCCTTGTTCTTCGACCAGC-3’

In brief, both side testes of a fish were used to make a sample for total RNA extraction and three such samples were made for each reproductive phase. Total RNA was extracted using TRI reagent (Sigma-Aldrich, USA), RNA integrity was estimated by Bioanalyzer (Agilent Technologies, USA) and concentration was measured using NanoDrop (ND-1000, NanoDrop Technologies, USA). Samples with RIN 5 or above were considered for cDNA preparation. Two microgram RNA of each sample was treated with DNase I (Thermo Scientific, USA) for 30 min to remove DNA contamination. DNase I was inactivated by heat denaturation at 70°C for 10 min in the presence of EDTA. Further, single-stranded cDNAs were synthesized using Avian Myeloblastosis Virus Reverse Transcription kit (Cat# K1622, Thermo Scientific, USA) following the manufacturer’s protocol. For qPCR, gene-specific primers were designed from their respective nucleotide sequences using Primer3 Input and Gene runner (Table 1). The efficiency of individual primer was checked using serial dilutions of testicular cDNA and amplification of single specific product was confirmed based on melt curve analysis. The qPCR reactions in samples run in triplicate were carried out in Real-time system CFX96 (Bio-Rad laboratories, USA) using SYBR Green Master Mix (Cat# 4367659, Applied Biosystems, USA). The reaction cycle consisted of the following steps: initial denaturation at 95°C for 10 min, 40 cycles of denaturation at 95°C for 30 s, annealing and extension at gene-specific temperature for 1 min, and a final dissociation step for melt curve analysis. Considering resting phase as reference, 2^-ΔΔct^ method was used to calculate relative fold change in expression of selected genes during preparatory, spawning and postspawning. Ribosomal 18s RNA was used as house-keeping gene for normalizing the expression values of target genes in each testicular sample.

### Statistical analysis

One-way analysis of variance (ANOVA) was applied to analyze significant variation in relative fold change of testicular mRNA expression for each gene during different reproductive phases. Newman-Keuls multiple range test was used to compare the means. Data are expressed as mean ± S.E.M (*P*< 0.05).

## Results

### Illumina paired-end sequencing and *de novo* assembly

RNA extracted from testicular samples of four reproductive phases R, P, S and Ps had A_260_/A_280_ ratios ranging from 1.8–2.1 and RIN values of 7, 7.4, 8.5 and 8.2, respectively (S1 Table). RNA sequencing on Illumina Hiseq 2000 platform yielded 100 bp reads from both ends of each cDNA fragment. The data generated 41.94 million reads (10.2 GB) for sample R, 47.51 million (11.55 GB) for sample P, 61.81 million (15.03 GB) for sample S and 44.45 million (10.81 GB) for sample Ps (S1 Table). After trimming the adapters and removing low quality bases, processed reads of 40.03 (9.61 GB), 45.01 (10.79 GB), 58.6 (14.05 GB) and 42.19 (10.12 GB) million were obtained for samples R, P, S and Ps, respectively (S2 Table). This reduced the percentage of non-ATGC characters (0.03–0.3%) in the processed reads. Thereafter, transcriptome assembly for samples R, P, S and Ps generated 154557, 158650, 204966 and 178121 contigs and 105526, 105169, 122964 and 106544 transcripts, respectively (S3 Table). The maximum contig length and transcript length for sample of each reproductive phase were 33888 and 43967 bp (sample R), 31822 and 66285 bp (sample P), 13462 and 36569 bp (sample S), and 17866 and 40661 bp (sample Ps), respectively. Length distribution of the assembled transcripts revealed that 789 transcripts for sample R, 1895 for sample P, 1928 for sample S and 1729 for sample Ps were ≥ 10 Kb in size. The total testicular transcriptome generated by clustering of transcripts from the four reproductive phases provided 210833 transcripts.

### Functional annotation of transcripts

**Fig 1:**
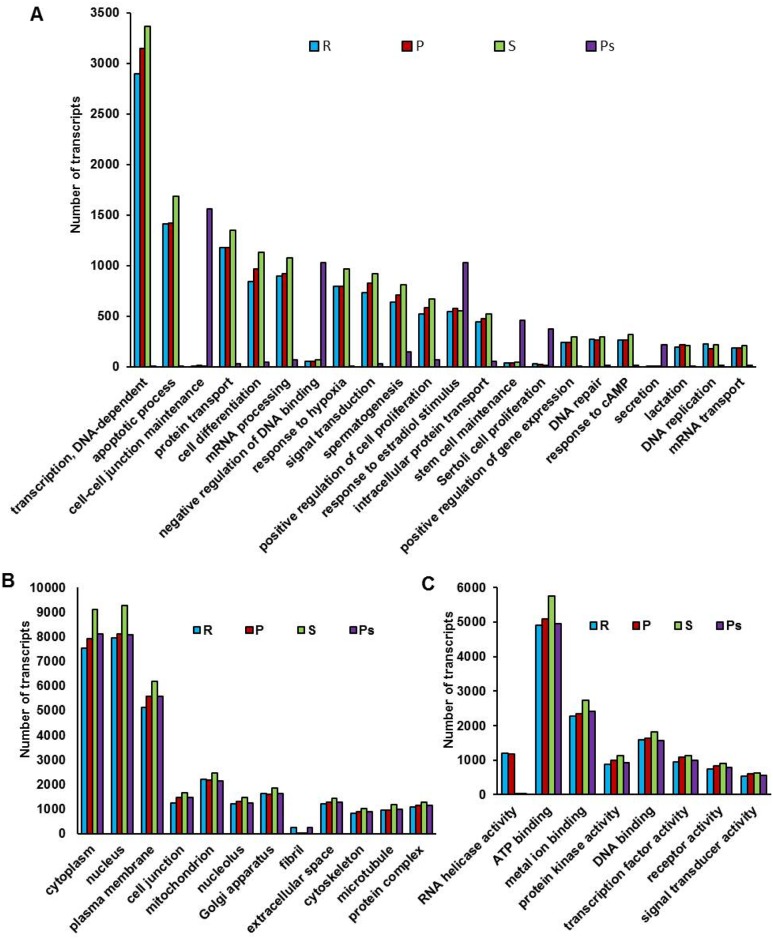
Histogram representation of gene ontology classification of transcripts from different reproductive phases. GO classification of testicular transcripts from different reproductive phases (resting: R, preparatory: P, spawning: S and postspawning: Ps) into various subcategories under Biological process (A), Molecular function (B) and Cellular component (C).

GO classification based on *R*. *norvegicus* protein database assigned the transcripts of four testicular samples to 5448, 1910 and 845 subcategories under BP, MF and CC, respectively. Majority of the subcategories under BP had lowest transcript number during postspawning phase that increased considerably in resting and reached the highest during spawning phase (Fig 1A). However, transcript number for some of the BP subcategories such as “cell-cell junction maintenance”, “negative regulation of DNA binding”, “response to estradiol stimulus”, “stem cell maintenance”, “Sertoli cell proliferation”, and “secretion” were appreciably high in postspawning as compared to other reproductive phases. Under MF, all the subcategories except “RNA helicase activity” showed least number of transcripts during resting, subsequent rise in preparatory and maximum during spawning phase (Fig 1C). A similar trend with highest transcript number in spawning and lowest in resting phase was found in most of the subcategories under CC (Fig 1B). Interestingly, transcript number for the subcategory “fibril” was high in postspawning and resting while extremely low in preparatory and spawning (Fig 1B).

**Fig 2:**
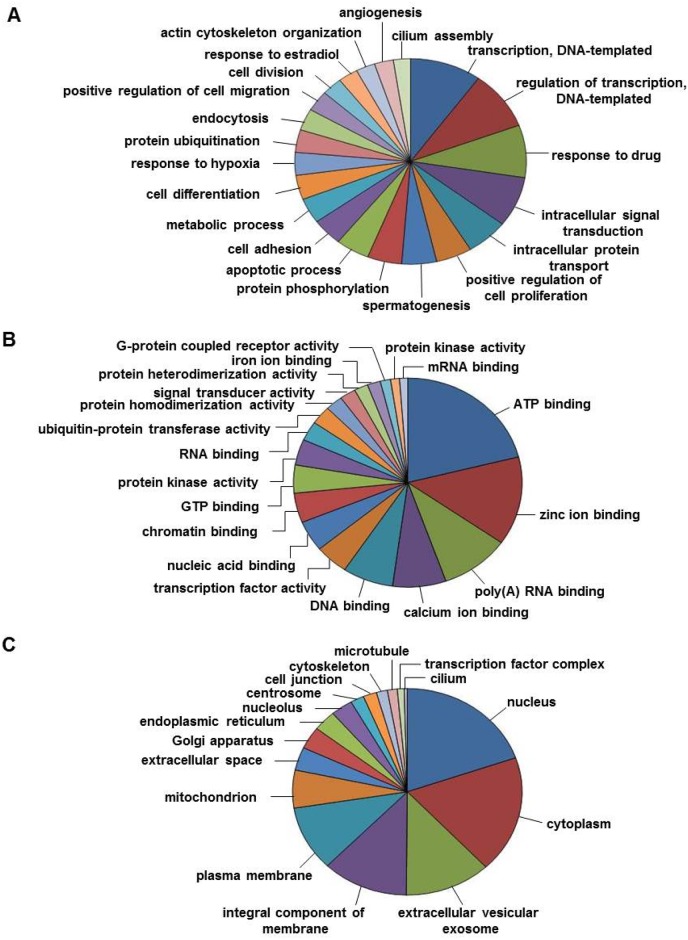
Pie diagram showing gene ontology classification of total testicular transcriptome. GO classification of total testicular transcriptome into various subcategories under: (A) Biological process (B) Cellular component (C) Molecular function.

GO classification of the total testicular transcriptome generated by clustering of transcripts from testis of four reproductive phases showed 8838, 2968 and 1187 subcategories under BP, MF and CC, respectively. Under BP category, “transcription”, “regulation of transcription, DNA-dependent”, “intracellular signal transduction” and “intracellular protein transport” were found to be the most represented subcategories (Fig 2A). In addition, significant number of transcripts was assigned to “positive regulation of cell proliferation”, “spermatogenesis”, “apoptotic process”, “cell differentiation” and “response to hypoxia”. In CC category, the subcategories “nucleus” and “cytoplasm” had the highest number of transcripts (Fig 2B). Under category of MF, subcategories related to different types of “binding” were frequently found along with “sequence-specific DNA binding transcription factor activity” and “protein serine/threonine kinase activity” (Fig 2C).

### Differential gene expression

**Fig 3:**
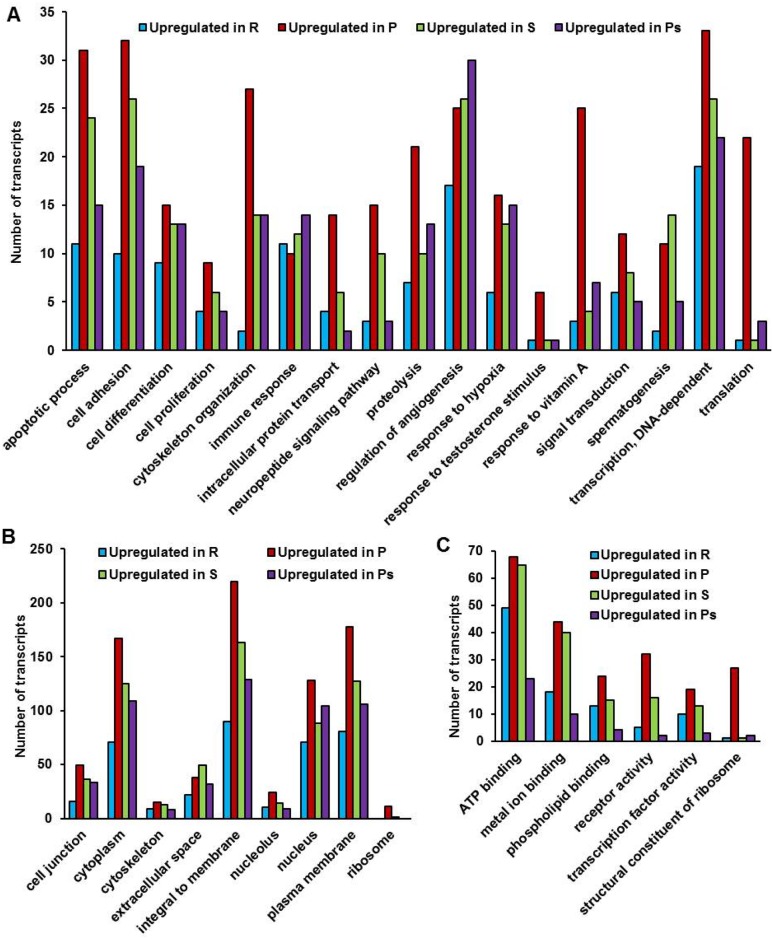
Histogram representation of gene ontology classification of upregulated testicular transcripts from different reproductive phases. Classification of upregulated testicular transcripts from different reproductive phases (resting: R, preparatory: P, spawning: S and postspawning: Ps) associated with testicular functions under GO subcategories of Biological process (A), Cellular component (B) and Molecular function (C). Upregulated transcripts were obtained from each set of clustered transcripts (set 1: samples R and P; set 2: samples P and S; set 3: samples S and Ps; set 4: samples Ps and R) based on expression fold change that was calculated considering the underlined sample in each set as reference.

Fig 3 presents a comparative picture of upregulated transcript numbers (sample R *vs* P, P *vs* S, S *vs* Ps and Ps *vs* R, considering the underlined sample as reference; S4 Table) under various functional subcategories of BP, CC and MF. A substantial increase in number of upregulated transcripts was observed for majority of the subcategories under BP during transition from resting to preparatory phase (Fig 3A) and thereafter transcripts displayed varying trends. The number of upregulated transcripts was maintained for “response to hypoxia” while a decrease was noted for “translation”, “response to testosterone stimulus”, “response to vitamin A”, “apoptotic process”, “cell adhesion” and “DNA-dependent transcription” during spawning. In contrast, an increase in number of upregulated transcripts was observed for “spermatogenesis” and “regulation of angiogenesis” until spawning and postspawning, respectively. Interestingly, upregulated transcript number for “immune response” decreased from resting to preparatory followed by a gradual increase during spawning and postspawning phases. Like BP, majority of the subcategories under CC showed maximum increase in number of upregulated transcripts during preparatory phase which subsequently decreased in spawning and postspawning (Fig 3B). Regarding MF, all the subcategories displayed a similar pattern for upregulated transcripts with highest number in preparatory followed by a decrease in spawning and postspawning (Fig 3C). It was interesting to note that upregulated transcripts for “structural constituent of ribosome” under MF and “ribosome” under CC were essentially present during preparatory phase.

**Fig 4:**
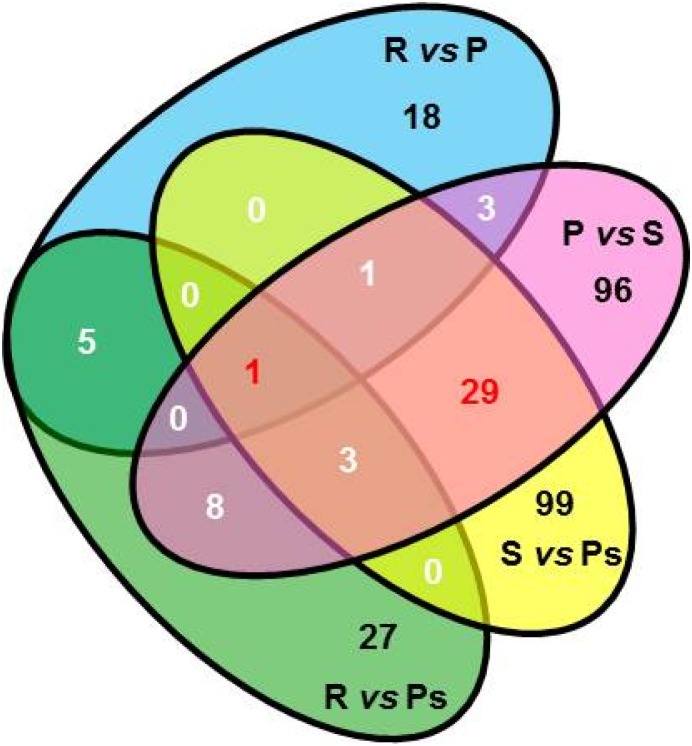
Venn diagram of testicular transcripts showing significant differential expression depending on reproductive phases (corrected *P* value < 0.05).

**Fig 5:**
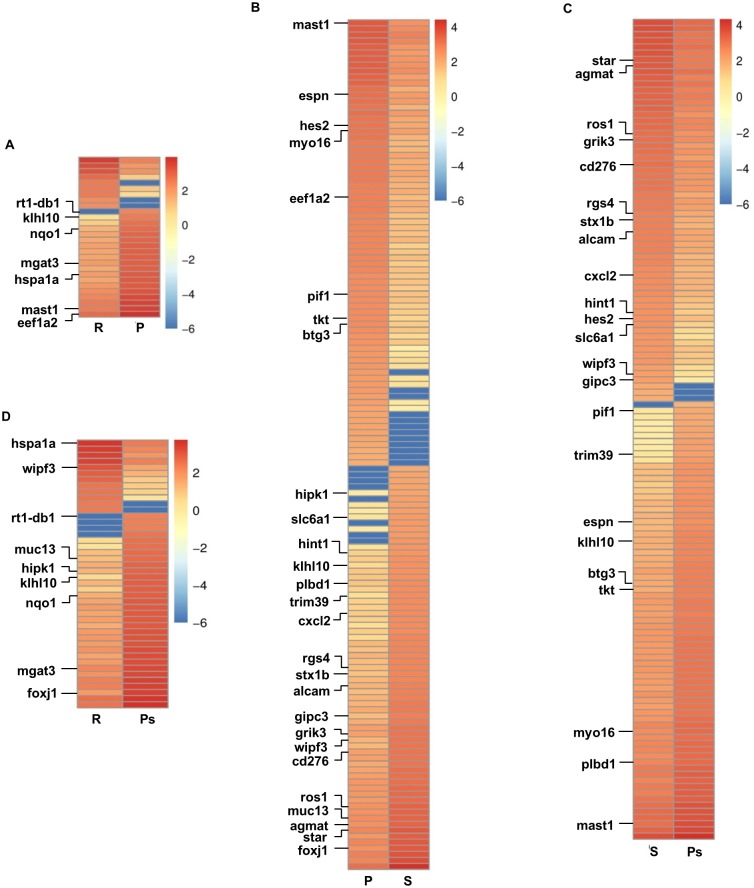
Heat map representation of significantly differentially expressed testicular transcripts. Shows testicular transcripts showing significant (corrected *P* value < 0.05) differential expression based on comparison between different reproductive phases (resting: R, preparatory: P, spawning: S and postspawning: Ps): (A) R and P, (B) P and S, (C) S and Ps and (D) R and Ps. Transcripts differentially expressed in more than two reproductive phases are labelled.

In addition, among transcripts showing significant (corrected *P* value < 0.05) differential expression, fifty transcripts were differentially expressed in more than two reproductive phases of which thirty were common to preparatory, spawning and postspawning (Fig 4). Throughout the reproductive cycle, the overlapping transcripts showed varying patterns of expression (Fig 5). A significant (corrected *P* value < 0.05) increase in expression of transcripts for *klhl10*, *rt1-db1*, *mgat3* and *nqo1* was observed in preparatory (R *vs* P, corrected *P* value < 0.05) that remained upregulated during spawning and postspawning. Similar upregulation in expression of transcripts for *mast1*, *hspa1a*, *eef1a2*, *hes2*, *btg3*, *pif1*, *myo16*, *espn*, and *tkt* was seen during preparatory phase, though their expression except for *hspa1a* significantly (P *vs* S, corrected *P* value < 0.05) decreased in spawning. Further, a set of transcripts (*wipf3*, *rgs4*, *star*, *gipc3*, *grik3*, *cd276*, *cxcl2*, *alcam*, *hint1*, *ros1*, *slc6a1*, *stx1b* and *agmat*) were profoundly expressed in spawning (P *vs* S, corrected *P* value < 0.05) and thereafter their expression declined during postspawning. On the other hand, upregulated expression of transcripts for *muc13*, *hipk1*, *foxj1*, *trim39* and *plbd1* during spawning remained high even in postspawning. Further, the analysis of expression levels for SC, LC and PMC specific genes during different phases of the testicular cycle revealed that only *amh*, a SC specific gene, showed significant upregulation in expression during postspawning phase (S *vs* Ps, corrected *P* value < 0.05, S5 Table). The other SC, LC and PMC specific genes, though detected in the testis, did not show any change in their expression along the testicular cycle (S5 Table).

### Validation of differential expression of genes

**Fig 6:**
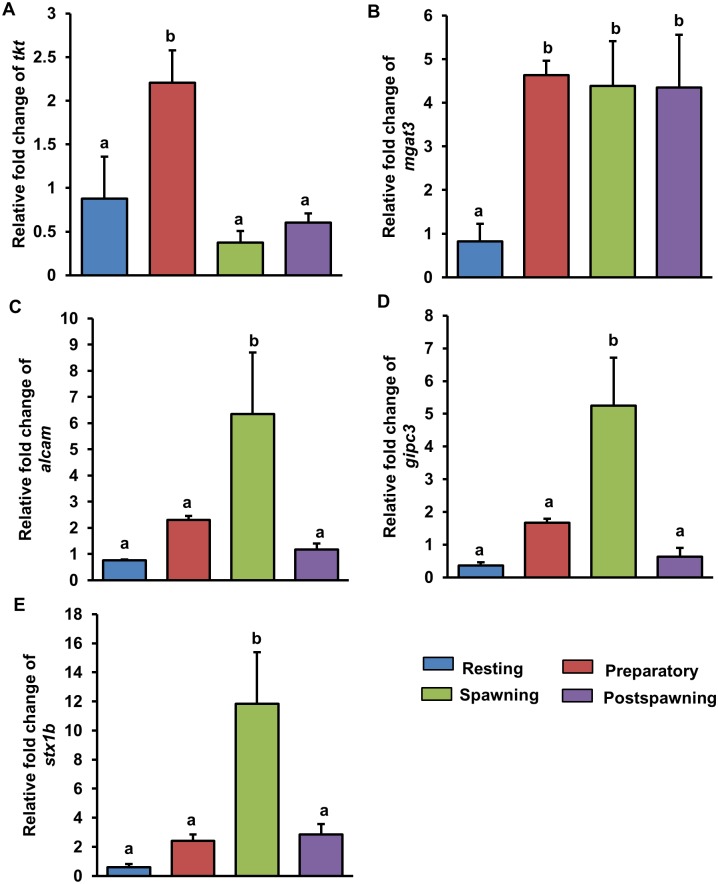
Showing expression fold change of some differentially expressed testicular genes *tkt* (A), *mgat3* (B), *alcam* (C), *gipc3* (D) and *stx1b* (E) along the reproductive cycle. The expression fold change of genes during preparatory, spawning and postspawning phases were calculated using expression values obtained during resting phase as reference. Ribosomal 18s RNA was used as the house-keeping gene for normalization of expression values. Three testicular samples were used for each reproductive phase (N = 3). Data represented as mean ± SEM were analyzed by one way analysis of variance (ANOVA) and compared by Newman-Keuls multiple range test. Groups with different alphabets (a-b) as superscripts show significant difference (*P* < 0.05).

The expression fold change of the selected testicular genes (*tkt*, *mgat3*, *alcam*, *gipc3* and *stx1b*) along the reproductive cycle showed significant (ANOVA, *P* < 0.05) variation depending on reproductive phases. The level of *tkt* and *mgat3* considerably (*P* < 0.05) increased during preparatory phase as compared to that of resting phase (Fig 6A and 6B). Thereafter, *tkt* expression in spawning phase declined (*P* < 0.05) to the level of resting and remained low until postspawning. Unlike *tkt*, a steady high expression of *mgat3* was recorded from preparatory to postspawning. In case of *alcam*, *gipc3* and *stx1b*, expression level though did not show any change until preparatory phase, a significant increase was observed during spawning phase (resting/preparatory phase *vs* spawning phase, *P* < 0.05; Fig 6C–6E). However, in postspawning, their expression declined to the level of resting/preparatory phase. These qPCR results showed a similar temporal expression pattern as observed following RNA-Seq analysis during different reproductive phases.

## Discussion

In the present study, comparative analysis of testicular transcriptome from different reproductive phases of spotted snakehead *C*. *punctatus* highlights the activation/repression patterns of several genes and their correlation with various structural and functional aspects of testis depending on its spermatogenic state. This study also provides the complete testicular transcriptome which is of particular importance as genomic resource for any species of *Channa* is not available.

### Comparative analysis of transcripts based on functional annotation

#### Total transcripts

Functional categorization of testicular transcripts from different reproductive phases showed high number of transcripts for majority of subcategories under BP, CC and MF during preparatory and spawning phases. The augmented spermatogenic activity during these phases might be correlated with marked increase in transcripts for major biological processes such as cell proliferation and differentiation, DNA repair, response to hypoxia, transcription, mRNA transport and processing, spermatogenesis, signal transduction, positive regulation of gene expression and protein transport. Similar correlation has been drawn in *Oncorhynchus mykiss* and *Mus musculus* wherein some of these biological processes were prominent during proliferation and differentiation of spermatogonia [19,27]. During postspawning, spermatogenic quiescence might be associated with decrease in transcripts for several biological processes and increase in transcripts for “negative regulation of DNA binding”. Further, enhanced transcript number for biological processes such as cell-cell junction maintenance, stem cell maintenance and SC proliferation could be implicated in restructuring of testis required for initiation of next testicular cycle. The restructuring of testis during postspawning is evident by an increase in transcripts for an extra-cellular matrix (ECM) component “fibril” as ECM components have been suggested to be involved in reorganization of seminiferous tubules [28]. Number of transcripts for “fibril” remained high during resting phase probably due to marked decrease of germ cells as compared to somatic cells (PMC and SC) that are reported to secrete ECM components [28]. In our earlier study in *C*. *punctatus*, estradiol-17β has been suggested to be involved in regression of testis, stem cell renewal and spermatogonial proliferation during postspawning phase [24]. Also, estradiol-17β is implicated in initiation and maintenance of spermatogonial stem cell proliferation in *Anguilla japonica* [29]. These reports substantiate our observation in the present study where number of transcripts for “response to estradiol stimulus” was highest during postspawning. Among molecular functions, high level of RNA helicase activity during preparatory phase in *C*. *punctatus* suggests an increase in translational activity as RNA helicases have been reported to play important role in ribosome biogenesis, pre-mRNA splicing and translation [30]. However, the reason for its increase in resting phase is not clear.

#### Differentially expressed transcripts

An upsurge in differentially expressed testicular genes has been reported in parallel to advancement of spermatogenesis in mammals [2,27]. A similar increase in upregulated transcripts concomitant to heightened spermatogenic activity was observed during preparatory phase in *C*. *punctatus*. Among these, an increase in transcripts specific to “proteolysis”, “signal transduction” and “intracellular protein transport” under BP is in accordance to a report in mice in which these transcripts are seen to be preferentially expressed in spermatids [31]. Moreover, an increase in number of upregulated transcripts for “translation”, “ribosome” and “structural constituent of ribosome” under BP, CC and MF, respectively, in preparatory phase suggests the amplification of protein synthesis during this period in *C*. *punctatus*. The rise in upregulated transcripts for “response to vitamin A” evidenced during preparatory phase in spotted snakehead indicates the involvement of vitamin A in proliferation and differentiation of spermatogonia. This is in agreement to a report in mammals where positive role of vitamin A in synchronization of spermatogonial differentiation and meiotic entry is documented [32]. An upregulation of transcripts for “response to hypoxia” during preparatory, spawning and postspawning might be the consequence of increased hypoxia due to escalation in cell proliferation. Our assumption relies on the report of Marti and colleagues [33] where direct relationship has been demonstrated between hypoxia and cell proliferation in mice testis. Further, hypoxia has been reported to induce angiogenesis [34], thus supporting our current observation of gradual increase in upregulated transcripts for “regulation of angiogenesis” from preparatory to postspawning phase. The present study also noted an increase in number of upregulated transcripts for “spermatogenesis” during preparatory and spawning phases probably owing to abundance of spermatocytes, spermatids and spermatozoa. A similar observation is reported in *O*. *mykiss* where genes grouped under biological process “spermatogenesis” are suggested to be expressed by meiotic/post meiotic germ cells [19].

### Upregulated transcripts and spermatogenic events

#### Preparatory phase

The present endeavor identified several transcripts significantly upregulated during preparatory phase suggesting their involvement in initiation and maintenance of spermatogenesisin *C*. *punctatus*. The cell-specific localization of some of these transcripts and their role in testicular development and spermatogenesis has been studied in mammals. *klhl10* and *mast1* are reported to be expressed in spermatids and associated with spermiogenesis [35,36]. A SC-specific gene *espn* has been implicated in the formation of blood-testis barrier [37]. *nqo1* is shown to be expressed in LCs [38] and its increased level from infancy to adulthood has been associated with testicular development [39]. Expression of heat shock protein *hspa1a* in PMC, SC as well as spermatogonia [40] and its downregulation in case of azoospermia [41] indicate the involvement of *hspa1a* in sperm development.

In addition to these transcripts, expression of *hes2*, *eef1a2*, *rt1-db1*, *mgat3*, and *tkt* have been detected in mammalian testis [42–46] though their cell-specific localization and definite testicular function is still unexplored. The upregulation of Notch effector gene *hes2* during preparatory phase in spotted snakehead *C*. *punctatus* indicates the role of Notch signaling in fish spermatogenesis as reported in mammals [47–49]. Further, current observation of increased *eef1a2* expression is substantiated by a report where eEF1A has been shown to be indirectly associated with protein synthesis and cell proliferation throughY-encoded testis-specific protein [43]. In addition, we observed upregulated expression of *tkt* and *mgat3* that encode enzymes involved in pentose phosphate pathway (PPP) and biosynthesis of glycoproteins, respectively, during preparatory phase when steroidogenic and spermatogenic activity markedly increases. In mammals, PPP has been found to be active in germ cells [50]. Further, PPP is known to facilitate steroidogenesis and nucleic acid biosynthesis through production of steroidogenic cofactor nicotinamide adenine dinucleotide phosphate and ribose 5-phosphate, respectively [51]. In fishes, the importance of glycoprotein conjugates in testicular cells has been documented where they have been associated with cell cycle, cell adhesion, proliferation, apoptosis and sperm maturation [52]. These facts provide the basis to assume the involvement of *tkt* and *mgat3* in upregulation of spermatogenic and steroidogenic activity during preparatory phase in *C*. *punctatus*. In our study, increased expression of *rt1-db1* during preparatory phase points towards its involvement in regulation of testicular functions though correlation between this immune response gene [53] and testicular functions is lacking in vertebrates. It is noteworthy that the current study demonstrates the expression of *pif1*, *btg3* and *myo16* for the first time in testis of a vertebrate. *pif1* and *myo16* are reported to be involved in cell cycle progression [54,55] whereas *btg3* has anti-proliferative action [56] and their upregulation during preparatory phase in *C*. *punctatus* suggests their role in maintaining cell homeostasis.

#### Spawning and postspawning phase

The identification of several testicular transcripts upregulated during spawning and postspawning phases in spotted snakehead *C*. *punctatus* are of vital importance to enrich our understanding of genes regulating spermatogenic and steroidogenic processes occurring during these phases of the testicular cycle. Transcripts known to be associated with sperm maturation (*ros1* [57], *foxj1* [58]), sperm transport and motility (*muc13* [59], *hint1* [60]), and acrosome reaction (*stx1b* [61]) increased significantly during spawning indicating their role in maturation, spawning of spermatozoa and fusion of gametes in *C*. *punctatus*. In addition, in the current study, an increase in expression of (a) *gipc3* known to promote luteinizing hormone action [62] and consequently production of maturation inducing steroid [63], (b) *grik3* reported to be associated with LC proliferation [64] and (c) *star* essential for steroid biosynthesis in LC and SC [65] suggest the importance of these genes in sex steroid biosynthesis and somatic cell proliferation in testis of *C*. *punctatus* during spawning. Among other upregulated genes of spawning phase, *rgs4*, *alcam* and *cxcl2* have been associated with gonadal stem cells [66–68] while *slc6a1* and *hipk1* have been reported in spermatids and spermatozoa [69,70]. Nonetheless, it is difficult to decipher the precise role of these genes in regulation of testicular events attributed to spawning phase in *C*. *punctatus*. To our knowledge, expression of *agmat* in testis is not reported in literature though its upregulation was observed during spawning in the present study. *agmat* encodes enzyme agmatinase that converts agmatine to putrescine [71]. Further, level of putrescine has been shown to increase in elongated spermatids of rooster [72], indirectly indicating the role of *agmat* in spermatogenesis. During postspawning, restructuring of seminiferous lobules includes apoptosis of remnant germ cells and proliferation of spermatogonial stem cells to repopulate the testis. In the present study, upregulation of (a) *cd276* localized in SCs and involved in tissue remodeling [73], (b) *wipf3* expressed immensely at Sertoli-spermatogenic cell junctions [74], (c) *trim39* identified in testis [75] and shown to promote apoptotic signaling [76], and (d) *plbd1* identified as a marker of testicular germ cell tumor precursor [77] provide the basis to assume pivotal role of these genes in remodeling of testis during postspawning in *C*. *punctatus*. In addition, *amh* which is a SC specific gene and reported to be involved in maintaining immature state of testis in mammals [78], was upregulated during postspawning phase in *C*. *punctatus*.

## Conclusion

The present study for the first time reports *de novo* testicular transcriptome sequencing in *C*. *punctatus*, thus contributing to the genomic information of this fish which is economically important and widely cultured in the Indian subcontinent. The functional annotation of testicular transcripts in *C*. *punctatus* highlighted the various biological processes, molecular functions and cellular components that are important for regulation of steroidogenic activity and spermatogenic events ranging from proliferation and differentiation of spermatogonia to release of spermatozoa. In addition, expression profile of testicular transcripts depending on reproductive phases enabled the identification of numerous upregulated transcripts associated with various testicular activities from preparatory to postspawning. The dataset of annotated transcripts and detailed overview of differential testicular transcriptional activity in the current study will provide basis for further investigation on functional genomic research and molecular regulation of testicular cycle in teleosts. Also, findings of this study may help in increasing fish production by manipulating testicular genes found to be important in regulation of spermatogenesis.

## Supporting information

S1 Table RNA QC, raw reads QC and base composition report of testis belonging to four reproductive phases. (XLSX)

S2 Table Processed reads QC, base composition report and quality score value of individual bases of processed reads in testis of four reproductive phases. (XLSX)

S3 Table Detailed information regarding *de novo* assembly of processed reads belonging to testis of four reproductive phases into contigs and transcripts using Velvet and Oases, respectively. (XLSX)

S4 Table Upregulated and downregulated transcripts belonging to different reproductive phases. (XLSX)

S5 Table Expression profile of somatic cell-specific transcripts during different reproductive phases. (XLSX)
